# Leatherbacks Swimming *In Silico*: Modeling and Verifying Their Momentum and Heat Balance Using Computational Fluid Dynamics

**DOI:** 10.1371/journal.pone.0110701

**Published:** 2014-10-29

**Authors:** Peter N. Dudley, Riccardo Bonazza, T. Todd Jones, Jeanette Wyneken, Warren P. Porter

**Affiliations:** 1 Department of Zoology, University of Wisconsin–Madison, Madison, Wisconsin, United States of America; 2 Engineering Physics, University of Wisconsin–Madison, Madison, Wisconsin, United States of America; 3 Pacific Islands Fisheries Science Center, NOAA Inouye Regional Center, Honolulu, Hawaii, United States of America; 4 Department of Biological Sciences, Florida Atlantic University, Boca Raton, Florida, United States of America; University of Minnesota, United States of America

## Abstract

As global temperatures increase throughout the coming decades, species ranges will shift. New combinations of abiotic conditions will make predicting these range shifts difficult. Biophysical mechanistic niche modeling places bounds on an animal’s niche through analyzing the animal’s physical interactions with the environment. Biophysical mechanistic niche modeling is flexible enough to accommodate these new combinations of abiotic conditions. However, this approach is difficult to implement for aquatic species because of complex interactions among thrust, metabolic rate and heat transfer. We use contemporary computational fluid dynamic techniques to overcome these difficulties. We model the complex 3D motion of a swimming neonate and juvenile leatherback sea turtle to find power and heat transfer rates during the stroke. We combine the results from these simulations and a numerical model to accurately predict the core temperature of a swimming leatherback. These results are the first steps in developing a highly accurate mechanistic niche model, which can assists paleontologist in understanding biogeographic shifts as well as aid contemporary species managers about potential range shifts over the coming decades.

## Introduction

Ecological niche modeling analyzes a set of environmental conditions in a location to determine the likelihood or possibility of a species’ persistence. It is an important tool to answer the fundamental question of where a species can exist. Throughout the next century, climate change will likely shift many species’ ranges [1]. In addition to causing these range shifts, climate change makes predicting species future ranges difficult because it introduces regionally novel sets of abiotic conditions. With these novel circumstances, forming an empirical niche map (based on species presence/absence data and a statistical model) requires complex statistical methods and may still be inappropriate or inaccurate [2], [3]. Under climate change conditions, a more appropriate niche mapping approach may be mapping the fundamental niche [4] using a biophysical mechanistic niche model. A biophysical model permits any combination of abiotic conditions, novel or otherwise, provided the model can accurately represent how they affect the organism [5].

While the biophysical niche model approach is successful with terrestrial animals [6], [7], it is more difficult to implement with aquatic organisms. The main reasons for this difficulty are the strong links among heat transfer, metabolic rate and aquatic motion [8], and the difficulty in measuring each of these. First, while it is possible to measure metabolic rate for some aquatic animals (smaller tractable species), it is difficult or impossible to measure it for others (large species that do poorly in captivity). Second, the complex interaction between thrust and drag on a deforming body makes it difficult to quantify a moving animal’s useful work [9]. Finally, determining how rapidly a self-propelling, aquatic organism loses metabolic waste heat further complicates making a biophysical niche map.

Many methods attempt to address some of these difficulties. Trained live animals can fly/swim in a wind tunnel/flume or while tethered. The animals may wear pressure sensors [10], [11] or temperature sensors [12], [13]. There are also non-contact methods such as filming particle flow around an animal and calculating force with software [14], using cameras to measure reference point movement [15], [16], or using thermal cameras to measure temperature profiles (not possible for aquatic animals) [17]. In addition to logistical and ethical consideration of live animal trials, all these methods have limited resolution and cannot decouple thrust and drag.

Modern computational fluid dynamics (CFD) can address many live animal experiments’ shortfalls while giving biophysical niche models necessary information to make accurate predictions. CFD is now capable of simulating drag [18] or heat transfer [19] of static animals and even thrust and power of undulating swimmers [20]. Here we use a novel CFD technique to measure the force a swimming neonate leatherback sea turtle (*Dermochelys coriacea*) produces and the power and heat flux of a swimming juvenile leatherback. We then use finite volume modeling to determine the juvenile’s internal heat transfer accounting for complex physiological issues such as an insulating layer’s varying thickness, vasoconstriction and dilation, and counter current heat exchangers. We compare our results to tethered swimming neonate and juvenile turtles. Further, we show that, by using only an allometric equation for resting metabolic rate (RMR) and the turtle’s flipper beat frequency, our methods can accurately predict the juvenile’s core temperatures. To our knowledge, this work is the first use of a 3D dynamic CFD simulation to accurately predict the internal environment of an animal.

## Methods

### Neonate Experiments

Florida Atlantic University’s Institutional Animal Care and Use Committee, Protocol A10–18 approved work with live neonate turtles, which occurred under Florida marine turtle permit 073 to Jeanette Wyneken. For animal collection and captive care, please see Miller et al. 2009 and Jones et al. 2011 [21], [22].

To measure thrust of the swimming neonates, we attached individual turtles (n = 11, average mass 59.7 g) by tether to a Futek load cell (FUTEK Advanced Sensor Technology, INC. Irvine, CA USA, LSB200 JR S-Beam load cell, range 0–10 g, 5 pt. calibration with resolution 0.03% of total range). The turtles swam freely in a 35 cm×35 cm×35 cm (h, w, d) tank for 40 to 60 minutes with force recorded at 2.85 Hz. The tether ran through an eyelet just above the water surface (4 cm) and attached to the force balance above the tank. The tether and eyelet had the dual purpose of allowing the turtles to swim or dive in any direction without hitting the tank walls and bottom while also directing the turtle’s forward thrust in line with the compression/tension of the load cell. We recorded the turtles’ flipper motion using a high-speed camera (420 frames per second). The turtles’ changes in swimming direction through the trials allowed us to capture stroke video from lateral, posterior and anterior views.

### Neonate Swimming Simulations

**Figure 1:**
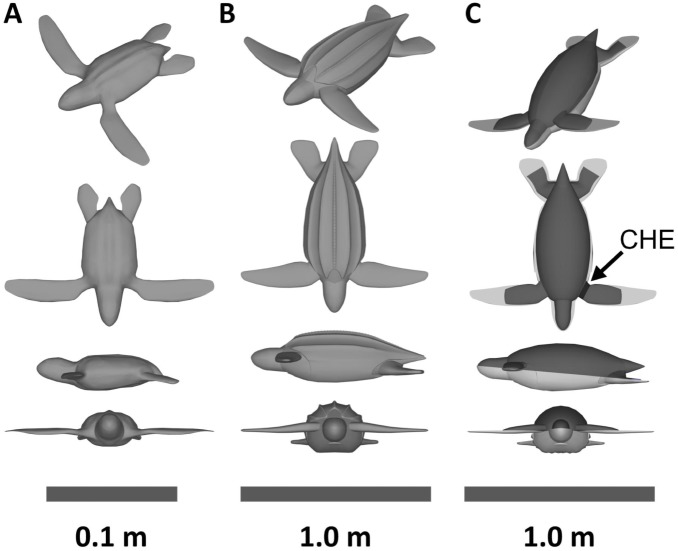
The 3D turtle drawings. (A) A 3D drawing of a neonate leatherback turtle. (B) A 3D drawing of a juvenile leatherback turtle. (C) A 3D drawing of a juvenile leatherback turtle with the top of the insulating shell virtually cut away. The figure shows the core zone in dark gray. The countercurrent heat exchanges (CHE) is highlighted on only one side in black.

**Figure 2:**
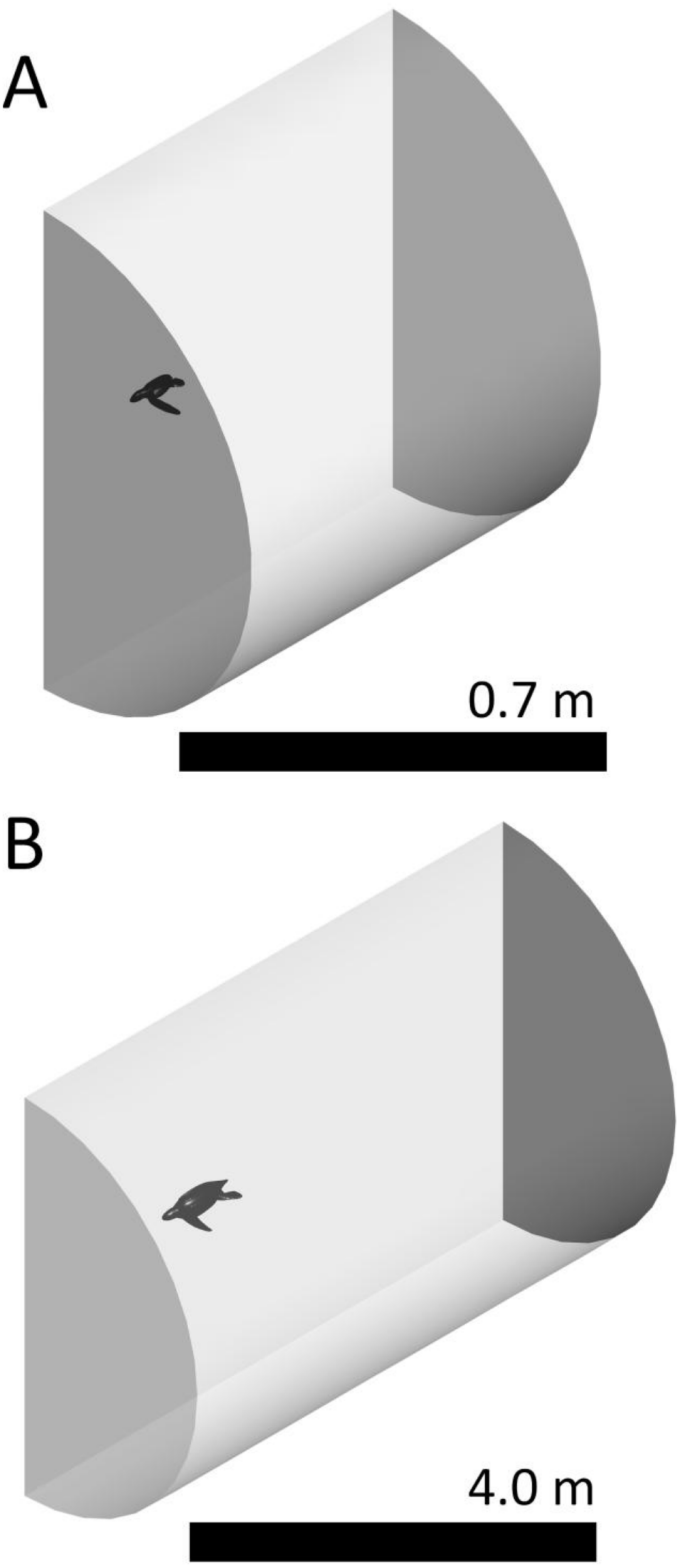
The volumes around the turtles. (A) The fluid volume that surrounds the moving, neonate turtle. The plane that divides the turtle had a symmetry boundary condition. This allows for reduction by half the computational fluid dynamics resource requirements. (B) The fluid volume that surrounds the moving, juvenile turtle. The plane that divides the turtle had a symmetry boundary condition.

**Figure 3:**
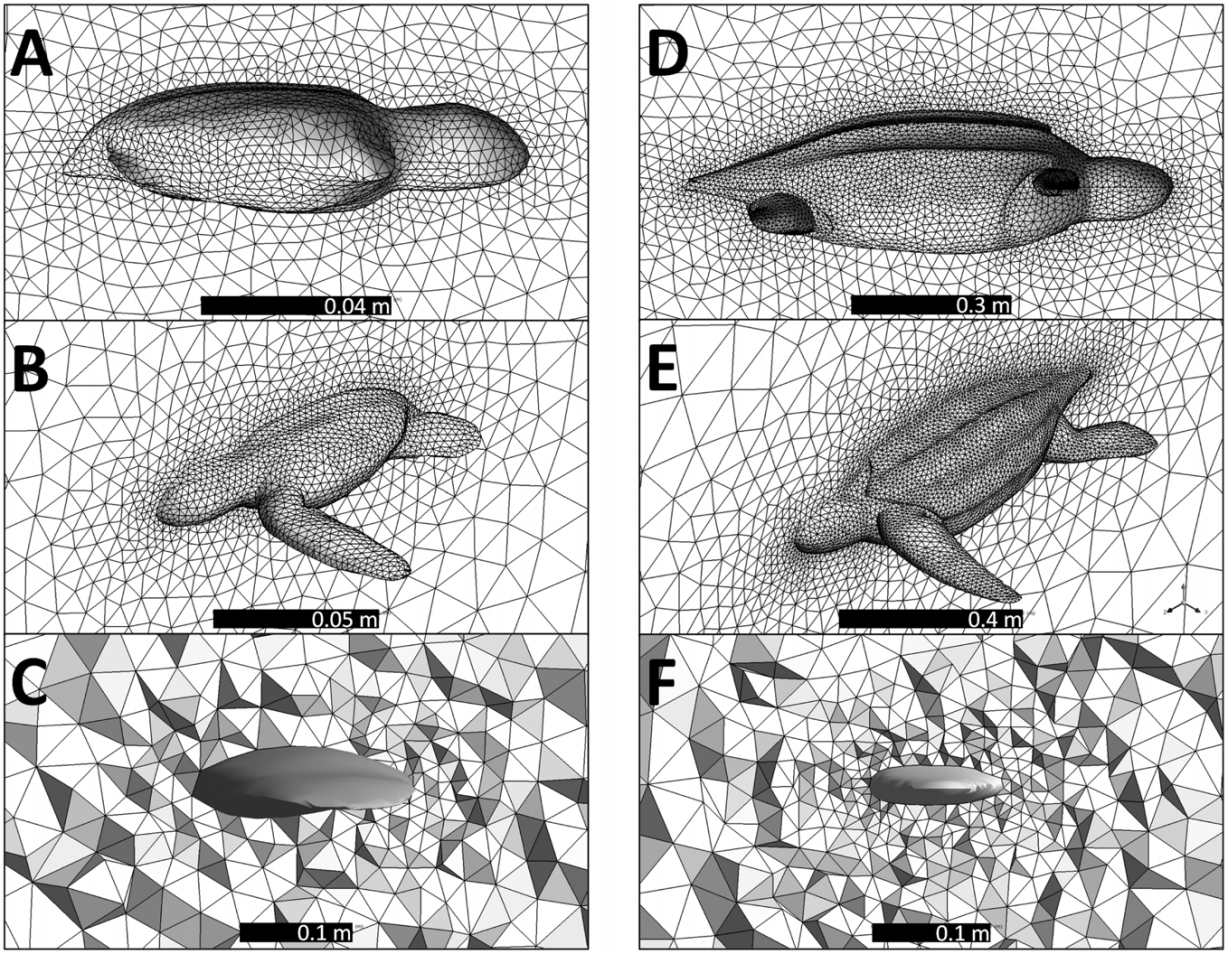
The mesh around the neonate model. Seen (A) from outside looking at the symmetry plane and (B) from inside the fluid volume looking towards the symmetry plane. (C) The mesh on a plane bisecting the neonate flipper at its midpoint. The mesh around the juvenile model seen (D) from outside looking at the symmetry plane and (E) from inside the fluid volume looking towards the symmetry plane. (F) The mesh on a plane bisecting the juvenile flipper at its midpoint.

Having measured thrust of a live neonate we now attempted to simulate this thrust. Using commercial 3D rendering software (ZBrush) we drew an anatomically realistic leatherback neonate in non-uniform rational basis splines (NURBS) format (Figure 1A). Drawing has the following advantages over scanning techniques: It is less expensive, it does not need a sample, it can be put in a format that CFD can use, can make an average morphology as opposed to only matching one sample, and it can produce any pose. The neonate had a curved carapace length (CCL) of 7 cm and would weigh approximately 60 g (morphology and summary data is in Table 1). We imported this NURBS model into ANSYS DesignModeler (ANSYS, Release 14.0) and enclosed one side of the model in a half cylinder, with the plane dividing the cylinder in half also dividing the turtle along the midsagittal plane. Enclosing only half the turtle and using a symmetry boundary condition on the plane bisecting the turtle increases computational efficiency. This half cylinder is the fluid region surrounding the turtle and had front, side and back buffers of 0.2 m, 0.4 m and 0.5 m respectively (Figure 2A). We set the buffers to be large enough to not influence the fluid interactions around the turtle. The half cylinder’s total volume (total fluid domain) was 0.3 m^3^. We imported this cylinder into ANSYS Meshing and constructed a tetrahedral polygon mesh throughout the entire fluid domain with 138,000 elements (Figure 3A, 3B, and 3C). We then imported this mesh into the CFD program ANSYS Fluent.

**Table 1:** A summary table of turtle morphologies and simulation run outputs.

Turtle/Run	Surface Area(m^2^)	Volume(m^3^)	Mass(kg)	AverageThrust(N)	Flipper HTC(W/K m^2^)	Body HTC(W/K m^2^)	Power(W)
Neonate	0.014	5.80×10^−5^	0.06	4.88×10^−3^	NA	NA	NA
Juvenile @ 6 BPM	0.89	0.036	36.9	0.060	464	260	0.043
Juvenile @ 19 BPM	0.89	0.036	36.9	0.832	775	303	0.371
Juvenile @ 25 BPM	0.89	0.036	36.9	1.540	952	305	0.904
Juvenile @ 29 BPM	0.89	0.036	36.9	2.070	1019	311	1.418

HTC is the heat transfer coefficient, and BPM is beats per minute.

We analyzed frames from the high-speed video using image analysis software (ImageJ version 1.46r) to measure the flipper motion. As we are attempting to simulate the neonate’s average thrust during the experiment, we analyzed eight sequences and used the average measures of the flipper motion. We wrote a supplemental program, which describes the turtles’ swimming motion we observed in the video. The flipper motion was smooth and divided into four zones. The 14% of the flipper closest to the shoulder was the transition zone from no motion to steady roll (flipper long axis moving dorsally to ventrally) and yaw (flipper long axis moving posteriorly to anteriorly). The next 22% of the flipper was the transition zone from steady roll and yaw to steady roll, yaw, and pitch (flipper twisting). The next 21% of the flipper was a zone of constant roll, yaw, and pitch. The last 43% of the flipper was the transition zone from steady roll, yaw, and pitch to roll, yaw, pitch, and bend (flexing of the flipper in the roll plane). Once we established the four zones, we constructed a program to make each zone move as seen in the video. We used products of rotation matrices to prescribe the motion of each node on the flipper surface. The roll matrices acted in the global coordinate system, the yaw matrices then acted in the rolled coordinate systems, the pitch then acted in the rolled and yawed coordinate system, and the bend then acted in the local flipper coordinate system. The stroke had four phases. There was a down stroke (0.384 s (length of phase), −1.15 radians of roll (positive is counterclockwise when looking at the turtle from the front), 0.45 radians of pitch (positive is counterclockwise when looking at the turtle from the side), −0.32 radians of yaw (positive is counterclockwise when looking at the turtle from the bottom), and 0.16 radians of bend (same sign convention as roll)), a turn at the bottom (0.199 s, 0.06 radians of roll, −0.71 radians of pitch, 0.16 radians of yaw, and −0.20 radians of bend), an up stroke (0.497 s, 0.96 radians of roll, −0.55 radians of pitch, 0.11 radians of yaw, and −0.16 radians of bend), and a turn at the top (0.163 s, 0.06 radians of roll, 0.81 radians of pitch, 0.07 radians of yaw, and 0.20 radians of bend). To prevent discontinuities in the model’s motion, each phase used 10% of its time to transition from the last phase.

As we expected turbulence, we used the *k-ω* shear stress transport (SST) viscosity model with a zero pressure inlet and outlet at the front and back of the turtle’s half cylinder enclosure (i.e. no flow velocity as the turtle in the experiment was tethered). We applied the no-slip condition applied on the turtle body and walls. The *k-ω* SST model provides accurate near wall performance of a *k-ω* model with the *k-ε* free-stream independence.. The fluid had the properties of seawater (density: 1015 kg/m^3^, specific heat: 4053 J/kg K, thermal conductivity: 0.6 W/m K and viscosity: 9.68×10^−3 ^kg/m s). We set the time step to 0.001 s and ran the simulation for 1442 time steps, which covers one full stroke sequence (i.e. one full period). To update the mesh during the motion, we used Fluent’s built-in spring-based smoothing (spring constant factor of 0.2 and a boundary node relaxation of 1) with local cell, local face and regional face remeshing. We calculated the turtle’s thrust by numerically integrating over each mesh node the instantaneous thrust in the swimming direction every 0.01 s as follows:
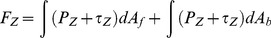
(1)where F_z_ is the instantaneous force in the swimming direction, P_z_ is the pressure component in the swimming direction, τ_z_ is the shear stress component in the swimming direction, A_f_ is the flipper area, and A_b_ is the body area. We then averaged that thrust over the entire run to get average thrust. We compared it with the experimental neonate force data.

### Juvenile Literature Data

To assess the ability of our method to model heat production and flux, we conducted four simulations to model turtles from Bostrom et al. [23]. They used a swallowed thermometer pill to measure the core temperature of tethered swimming leatherbacks while counting flipper stroke frequency and measuring ambient water temperature.

### Juvenile Swimming Simulations

To model juvenile internal heat production we first must simulate the power the turtle generates while swimming to determine an active metabolic rate. We drew an anatomically realistic juvenile leatherback in NURBS format using commercial 3D design software (Triple Squid Software Design, MoI V2.0) (Figure 2B). The juvenile had a curved carapace length (CCL) of 72 cm and would weigh approximately 37 kg. We imported this NURBS model into ANSYS DesignModeler and enclosed one side of the model in a half cylinder. The cylinder had front, side and back buffers of 1.9 m, 1.9 m and 3.8 m respectively (Figure 2B). The half cylinder’s total volume was 60.3 m^3^. We imported this cylinder into ANSYS Meshing and meshed the entire fluid domain with 195,000 tetrahedral elements (Figure 3D, 3E, and 3F). We tested a mesh with elements 1/8th the volume of our mesh during runs. We then imported this mesh in to ANSYS Fluent.

We used the same supplemental program to describe the juvenile turtle’s swimming motion that we used for the neonate turtle. We analyzed frames from publicly available video of leatherbacks freely swimming with ImageJ to measure the flipper motion. The videos were all of adults and had to contain clear views of the stroke. The flipper had the same four zones as the neonate. Again, the stroke had four phases. There was a down stroke (32.3% of stroke period, −0.95 radians of roll, 0.00 radians of pitch, −0.15 radians of yaw, and 0.00 radians of bend), a turn at the bottom and at the top of the stroke (17.0% of stroke period for each, 0.00 radians of total roll, ±2.40 radians of pitch, 0.00 radians of total yaw, and ±0.80 radians of bend), and an up stroke (33.7% of stroke period, 0.95 radians of roll, 0.00 radians of pitch, 0.15 radians of yaw, and 0.00 radians of bend). To prevent discontinuities in the motion, we programmed smooth linear transitions into the top and bottom phase. To correctly position the flipper, there was also a 0.5 s setup time. We used four of the flipper stroke frequencies from Bostrom et al. (6, 19, 25, and 29 beats per minute (BPM)) (2010).

The simulation used the *k-ω* SST model with a zero pressure inlet and outlet at the front and back of the turtle’s half cylinder enclosure (i.e. no flow velocity as the turtle in the experiment was tethered). The plane dividing the turtle had a symmetry boundary condition. We set the time step to 0.001 s and ran the simulation for one full period plus setup time. We also ran over two full periods to examine if the development of the flow would affect out results. In all analyses, we removed the initial 500 set-up steps. We calculated thrust by Eq. (1), and power by instantaneous numerical integration over each mesh node every 0.01 seconds with the following equation:
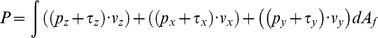
(2)Where P is the power the flipper puts into the water, p_z,x,y_ is the *z*, *x*, or *y* pressure component τ_z,x,y_ is the *z*, *x*, or *y* shear stress component v_z,x,y_ is the *z*, *x*, or *y* flipper velocity component, and A_f_ is the flipper area. To translate total work the turtle does on the water into expended energy, we used the aerobic efficiency of tortoise muscle (35% [24]). We calculated the flipper and body heat transfer coefficients by instantaneous numerical integration over each mesh node every 0.01 seconds by the following equation
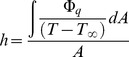
(3)where h is the heat transfer coefficient, Φ_q_ is the heat flux, T is the turtle’s skin temperature, T_∞_ is the free stream temperature, and A is the surface area. We averaged all those data over the entire run to get average values.

### Juvenile Internal Conduction Simulations

After calculating the power produced while swimming, we can now model the internal temperature of the active juvenile leatherbacks. Using literature information on insulating layer thickness, we drew an anatomically realistic core region of the adult leatherback in NURBS format [25]–[28]. We also included a countercurrent heat exchanger region in the flipper (Figure 1C) [29]. We set the insulating region density to that of blubber (980 kg/m^3^) [30]. We set the density of the core region to make the overall turtle neutrally buoyant (1035 kg/m^3^). This density is a reasonable number for a region comprised of muscle and organ tissue. We set the insulating region’s thermal conductivity to that of blubber (0.280 W/m/K) [31] and the countercurrent heat exchanger region’s thermal conductivity to that of dead tissue (0.531 W/m/K) (no blood flow) [32]. We described the core region’s thermal conductivity as a temperature dependent function going from 0.531 W/m/K at 17°C, to 0.622 W/m/K (conductivity of live muscle [32]) at 28°C, and to 50 W/m/K (a very high value to prevent the buildup of hot spots and unrealistic thermal gradients) at 47°C. The actual value of the thermal conductivity at 47°C had very little effect on the core temperature (a quadrupling of the thermal conductivity results in a decrease of less than 4% in the core temperature). This temperature dependence allows the model to simulate vasoconstriction and vasodilation given cold or hot temperatures. We set the insulating region’s heat capacity to 2.94×10^3 ^J/kg/K based on an average of lipid heat capacities [33] weighted by the lipid content of blubber [25] and water’s heat capacity weighted by the water content of blubber (33%) [34]. We set the heat capacity of the core region to that of live tissue (4.18×10^3 ^J/kg K) [32]. We used an allometric relation from Wallace & Jones (Eq. 4) to estimate the resting metabolic rate (RMR) for the juvenile turtle [35]. We scaled the RMR with core temperature based on a Boltzmann factor relation for reptiles (Eq. 5) [36]. This simulated turtle is treated as motionless and so to account for the increased convection that the movement of the flippers will cause, we set the thermal boundary condition on the outside of the flipper and body to be the heat transfer coefficients of each part obtained from the swimming simulation. We ran the internal simulations in ANSYS Fluent using its built in solid heat transfer capabilities.
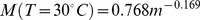
(4)where M (T = 30°C) is the metabolic rate at 30°C (W/kg) and m is the mass (kg)
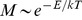
(5)where M is the metabolic rate, E is the average activation energy (0.76 eV), K is the Boltzmann’s constant (8.617×10^−5^ eV/K), and T is temperature. Core temperature of the simulated turtle varied based on region. From the simulations, we extracted the core region’s minimum, maximum, and average temperatures. We also extracted temperatures for which 87.5%, 75%, 25% and 12.5% of the turtle’s core volume was beneath (in other words, the temperatures bounding the 50^th^ and 75^th^ percentiles). We compared these temperatures to the measured internal temperatures for the live turtle in Bostrom et al. [23].

## Results

### Neonate

**Figure 4:**
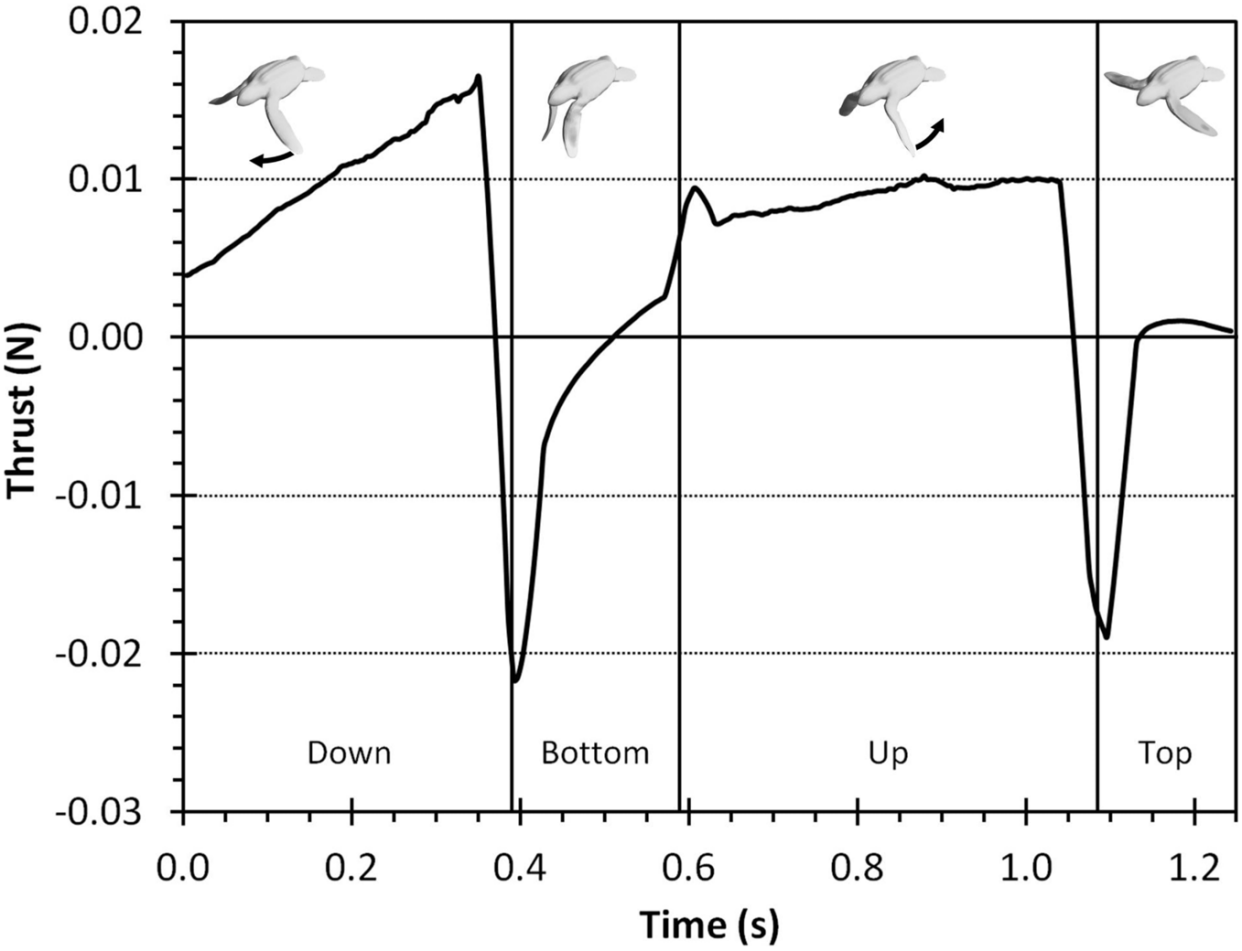
The thrust produced by our simulated neonate turtle as it goes through one stroke period. The vertical black lines demark the four different stroke phases (down, bottom, up, and top). The images at the top are clips from each respective phase.

**Figure 5:**
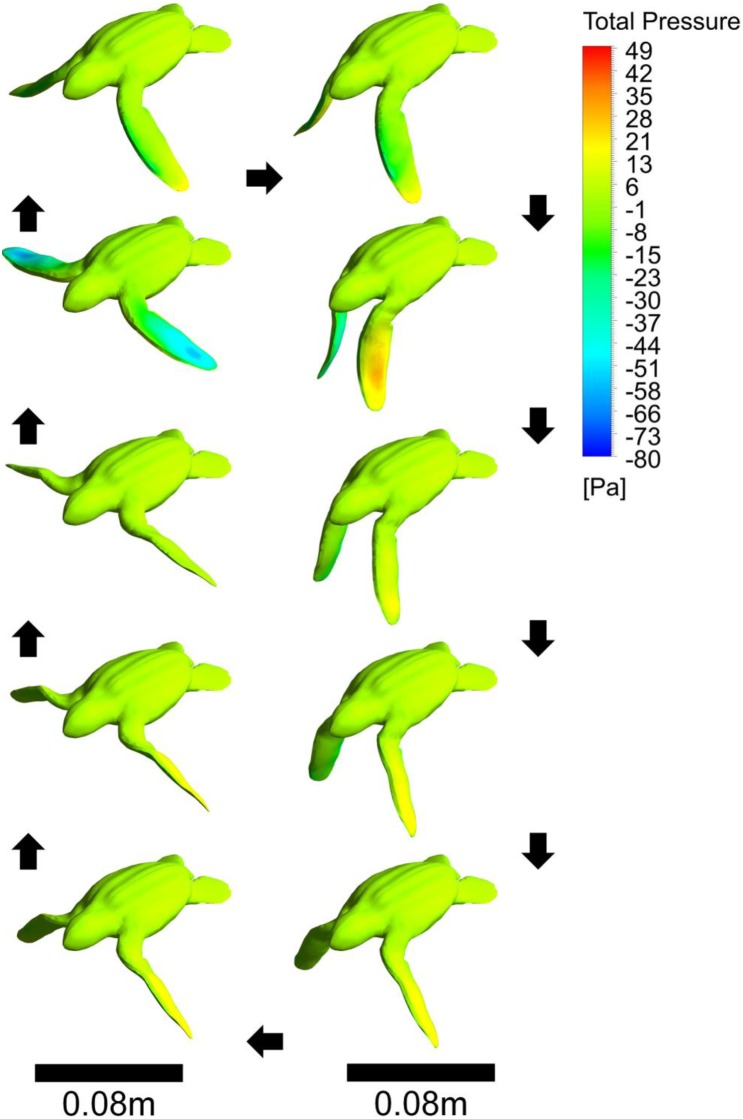
The pressure contours on the neonate turtle as it moves through one stroke period. Black arrows indicate the direction of the image sequence. This is an orthographic projection. The middle image in the left column represents one of the high negative thrust points recorded at the start of the bottom phase.

**Figure 6:**
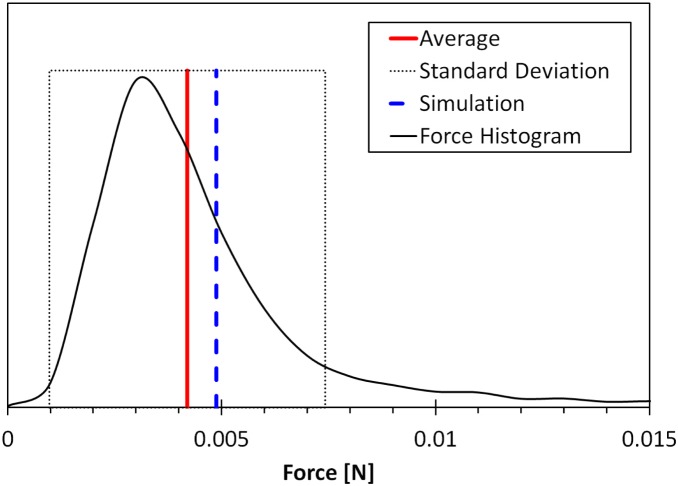
A comparison between measured and simulated neonate thrust. The width of the dotted box represents the experimental data’s standard deviation. Force histogram displays the distribution of force measurements recorded during the experiment.

The neonate simulated thrust profile shows positive and steadily rising thrust during the up and down phases of the stroke with initial pulses of negative thrust during the top and bottom phases (Figure 4). We see that the up and down phases provide the forward force to the turtle while the top and bottom phases are simply a necessity to setup the other phases and provide mostly reverse thrust. Figure 5 shows the pressure profile during the stroke. We see large pressures at the top and bottom turning points most likely owing to added mass (i.e. effect of accelerating fluid surrounding the flipper) Our simulated average force (0.00488 N) was within a quarter of the standard deviation with the experimental force measurements (0.004±0.003 N) (Figure 6).

### Juvenile

**Figure 7:**
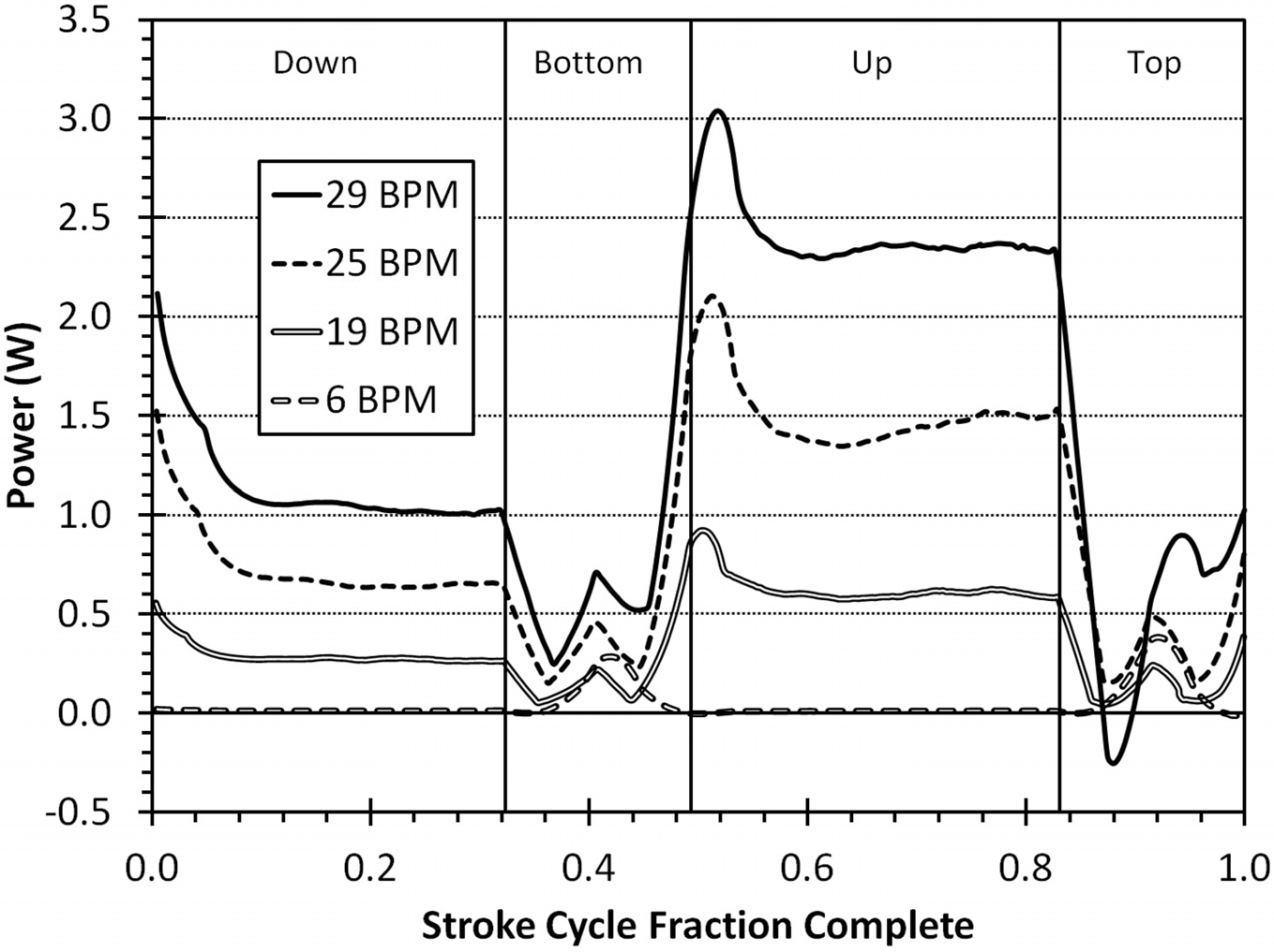
The power produced by our simulated juvenile turtles as they go through one stroke period. The vertical black lines demark the four different stroke phases (down, bottom, up, and top). Since each swimming frequency (beats per minute (BPM)) has its own period, the x-axis is percent of stroke completed.

**Figure 8:**
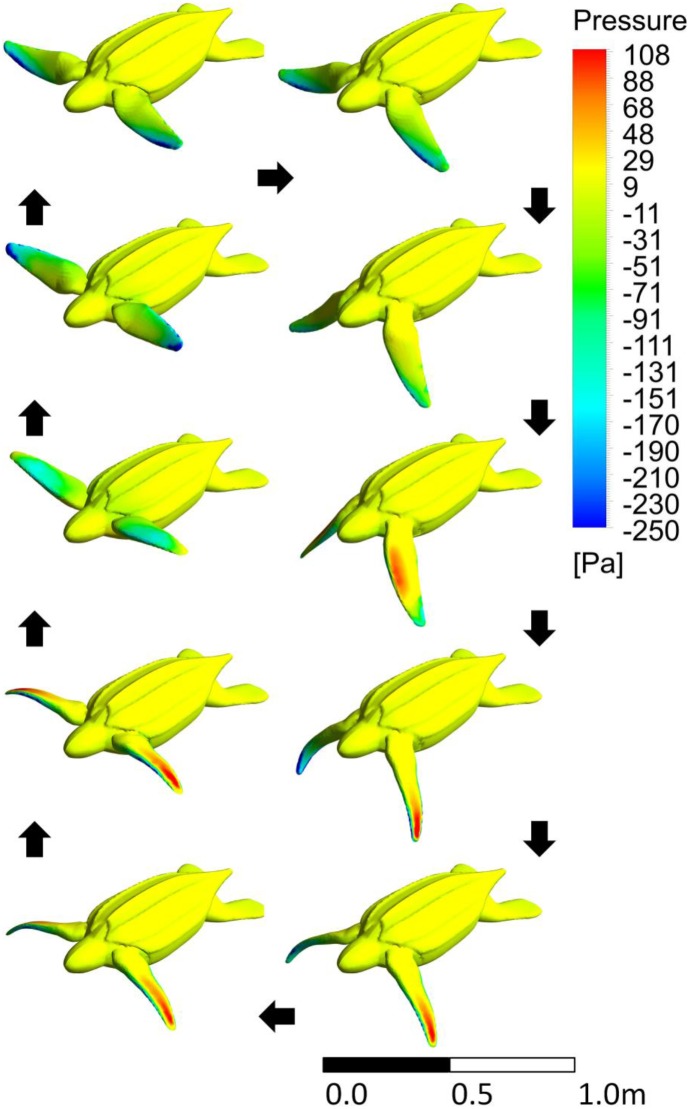
The pressure contours on the juvenile turtle as it moves through one stroke period. Black arrows indicate the direction of the image sequence. This is an orthographic projection. The third image in the left column represents one of the high negative thrust points recorded at the middle of the bottom phase.

**Figure 9:**
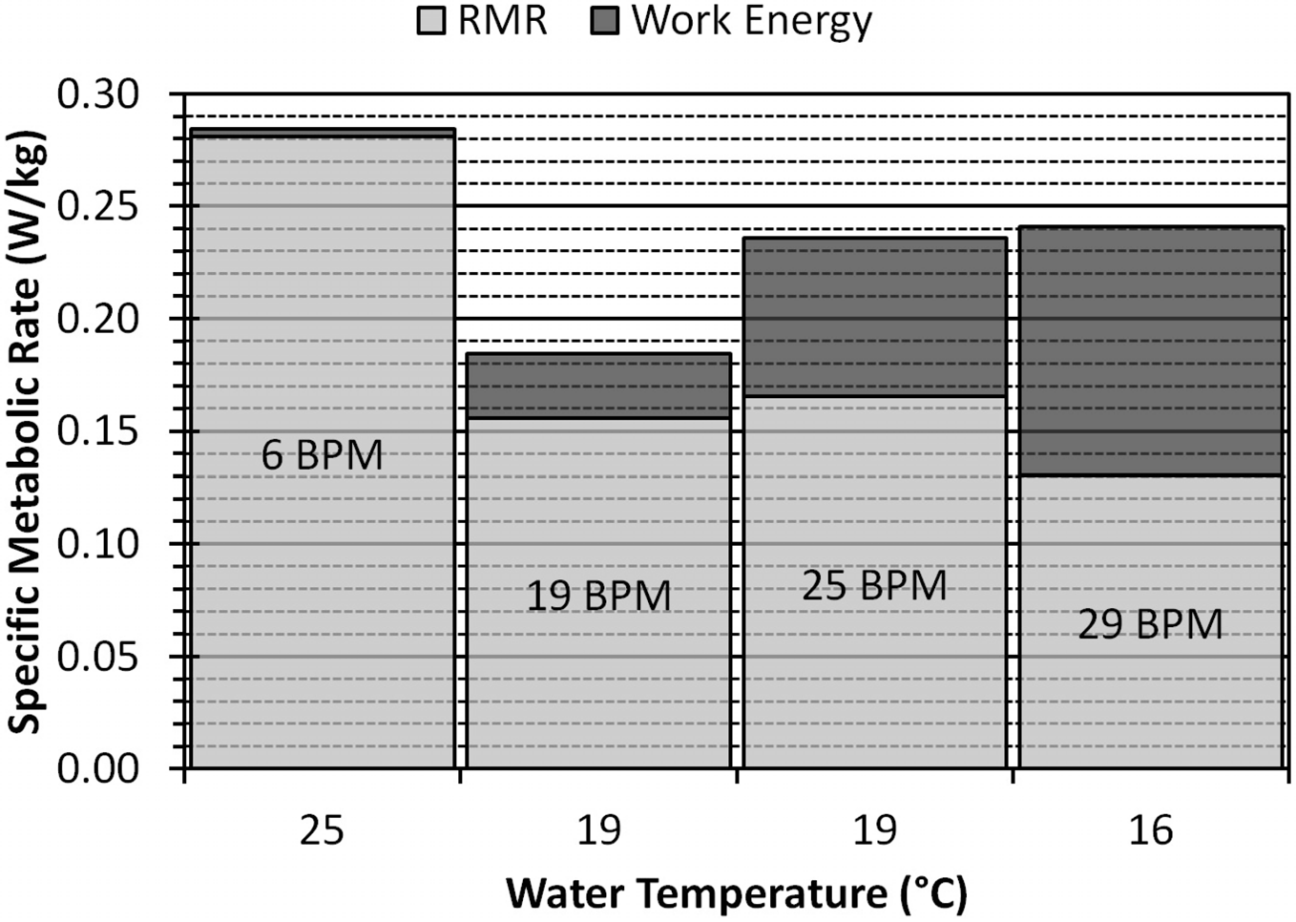
The proportion of specific metabolic rate owing to resting metabolic rate (RMR) and the metabolic cost of flipper movement (Work Energy). Each bar corresponds to a specific ambient water temperature (x-axis) and a specific flipper stroke frequency in beats per minute (BPM) (on bar labels).

Table 1 gives average flipper and body heat transfer coefficients, power and force for the juvenile simulation. The juvenile power profiles show a reasonable output with areas of positive thrust showing high power output (Figure 7). The up and down strokes have the highest power outputs while, like with the neonate, the top and bottom phases seem to simply set up the next phase of the stroke and add little power of their own. As would be expected, the higher the stroke frequency, the higher the force and power output. Figure 8 shows the pressure profile during the stroke. The highest-pressure regions are on the tips of the flippers. As these regions also have the highest velocities, they are responsible for the largest portion of the power. Again, we can see the effect of added mass on the flippers and they change direction. This added mass is responsible for added energy during the stroke, which does little useful work. In cooler waters, the metabolic cost of flipper movement (work energy) is responsible for a substantial portion of the total metabolic rate. In the warmest water, the work energy contributes little to the metabolic rate (Figure 9).

**Figure 10:**
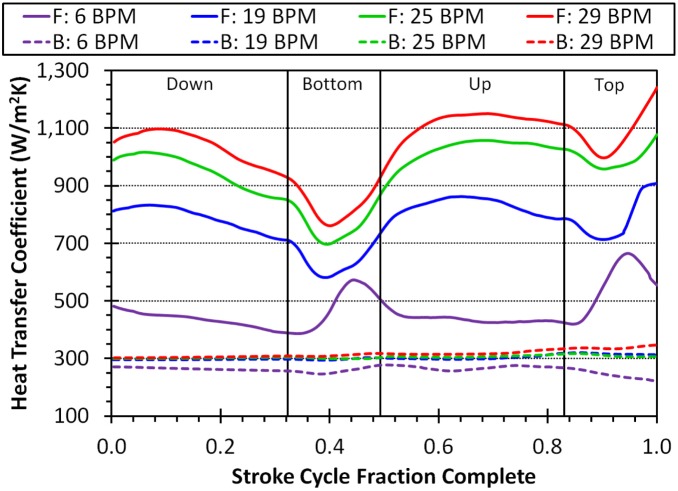
The heat transfer coefficients of our simulated juvenile turtles they go through one stroke period. B and F stand for the heat transfer coefficient on the body and flipper, respectively. The vertical black lines demark the four different stroke phases (down, bottom, up, and top). Since each swimming frequency has its own period, the x-axis is percent of stroke completed.

**Figure 11:**
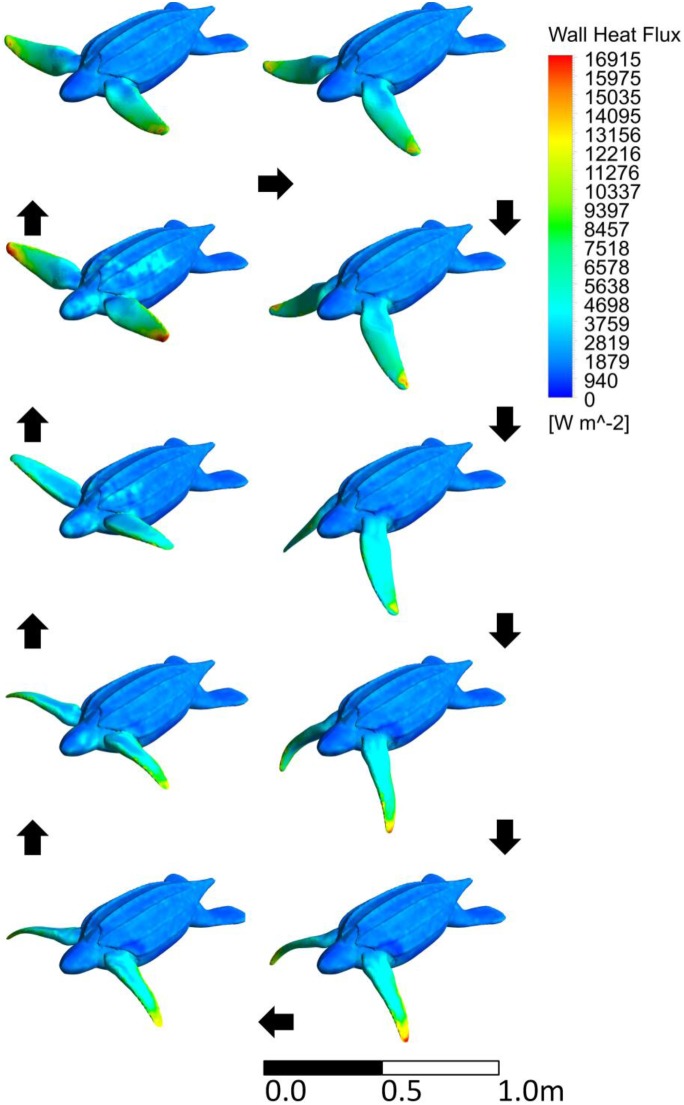
The turtle’s surface heat flux contours on the juvenile turtle as it moves through one stroke period. Black arrows indicate the direction of the image sequence. This is an orthographic projection.

**Figure 12:**
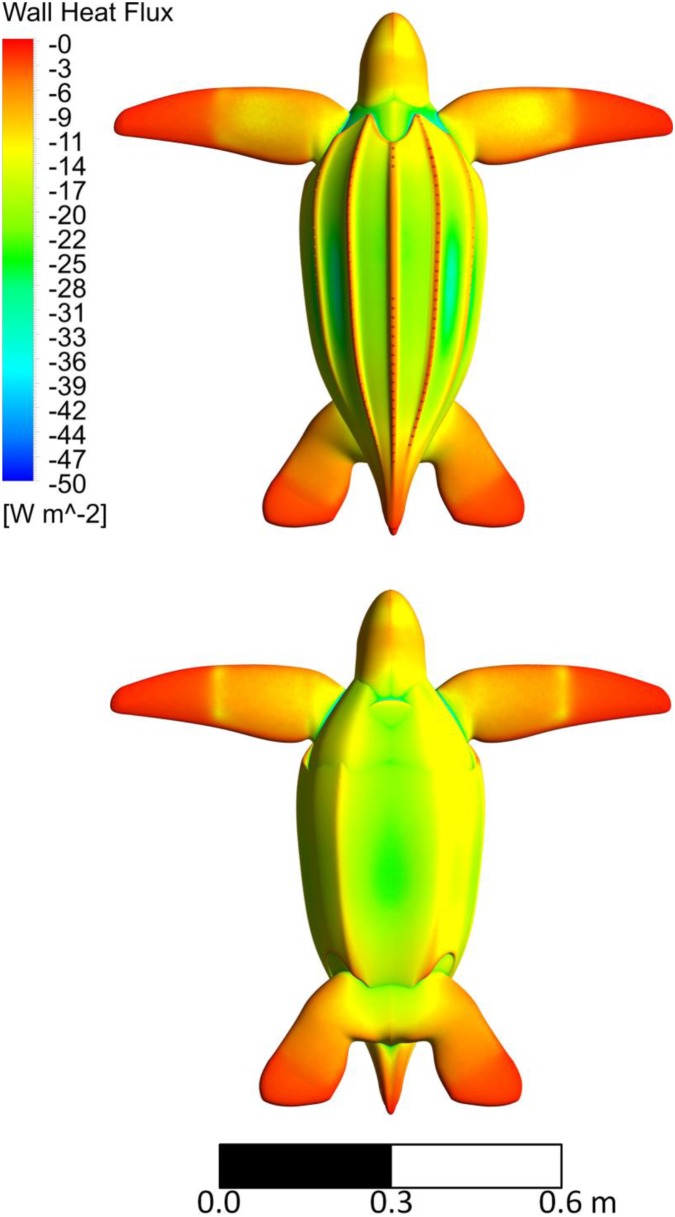
The heat flux contours on the internal conduction model of the juvenile turtle.

As predicted, the swimming juvenile heat transfer coefficients are much higher for the flippers than the body (Figure 10). The body heat transfer coefficient stays relatively constant over the stroke while the flipper’s varies with phase. For the slowest stroke, the turning motion during the top and bottom phase increase the flipper heat transfer coefficient where for a faster stroke the top and bottom phases decrease the flipper heat transfer coefficient. Figure 11 shows the higher heat flux on the flipper than the body. The internal conduction model shows high heat flux in the channels of the shell, high heat flux around the neck and shoulders, moderate heat flux on the upper flipper, and low heat flux on the flipper tips (Figure 12). Both the finer meshes and running over two stroke cycles made less than 5% difference in any flow parameter, which translates to an undetectable difference in our internal temperature results.

**Figure 13:**
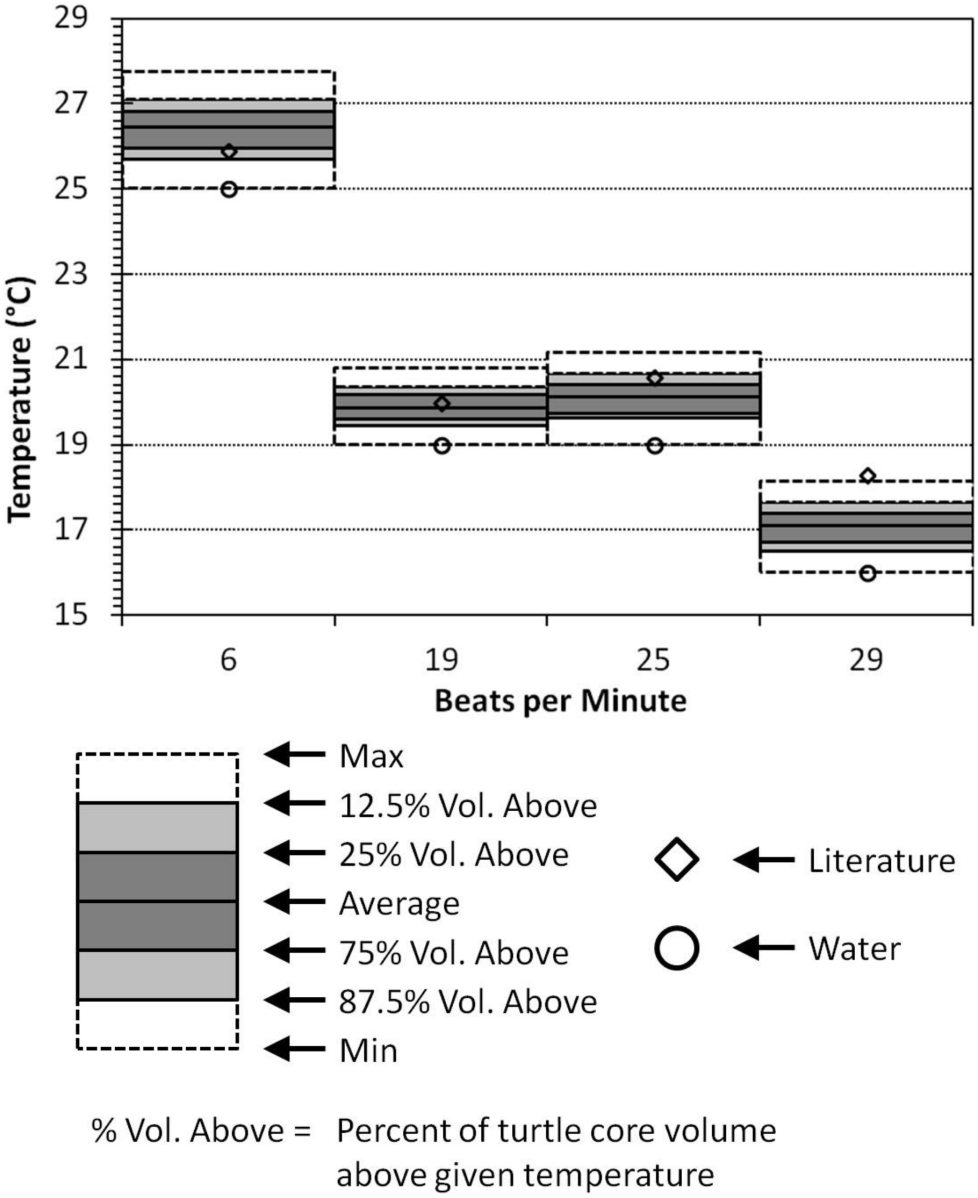
A comparison between the simulated and experimental internal temperatures. The dotted boxes contain the range of temperatures inside the simulated turtle’s core region. The light gray region bounds the 75^th^ temperature percentile of the turtle’s core volume and the dark gray box bounds the 50th Temperature percentile of the turtle’s core volume. Experimental data from literature [23].

Our simulated data are in good agreement with the internal temperatures of the real juvenile turtle [23]. Temperatures for the three highest stroke frequencies are within the temperature range containing 75% of the turtle’s core volume. The 29 BPM turtle’s core temperature is slightly outside maximum simulated core temperature (Figure 13).

## Discussion

The agreement between the average simulated neonate thrust and the average experimental neonate thrust gives us confidence that our methods can accurately model the dynamics of a sea turtle stroke. While the simulation is not an exact representation of the live turtle experiment, the differences are minimal. The main difference between our simulation and a live turtle is that we did not model the small translocations of the turtle’s body during the stroke cycle. The agreement between our juvenile temperature simulations and the live juvenile temperature records [23] gives us confidence that we can accurately model the internal environment of a turtle, particularly in warmer waters. Warmer conditions will be most important when considering climate change’s effects on leatherback’s tropical and subtropical range. The temperatures from Bostrom et al. contain more variation than the single reported number represents [23]. Examples of variation are two runs with the same water temperature and flipper stroke frequency, which had a 0.2°C difference in core temperature, and there was a case where a higher stroke frequency at the same water temperature resulted in a lower core temperature. The complex nature of this real animal system means that anomalies like these are inevitable, yet we are still able to model this system accurately at temperatures that are important for implementing this method into a mechanistic niche model.

This method and results have important applications in conservation. These methods can help construct a highly accurate mechanistic niche map of leatherback sea turtles and could potentially work for other marine organisms. These maps will show the potential range shifts under climate change. Such information allows planning for future species regional disappearances or appearances. As we integrate these methods into mechanistic niche mapping, other research groups should consider adopting more realistic animal geometries over simplified shapes when considering animal-fluid interactions [19].

In addition to conservation, these methods and results have important applications in the fields of biomechanics, physiology, fluid dynamics and engineering. There is much research going into the biomechanics of animal flight and swimming [37]. Our method of 3D, full motion animal CFD provides a novel robust tool to a field, which currently bases much of its research off static casts of fins and flippers. With regards to physiology, in addition to animals that propel themselves through fluid, there is much research needed on animals that actively cool themselves by manipulating the fluid around them [38]. Concerning CFD research, while CFD is used in understanding animal motion, currently, the only 3D, full animal motion CFD we were able to find was a 3D model with 2D motion [20]. The Liu et al. simulation also used proprietary software (as opposed to our commercially available software) and did not simulate heat transfer (1997). There is also active research and development on submersibles and drones that mimic animal propulsion (sea turtle example: [39]). Looking at engineering, by allowing researchers to use simulations rather than constructing many prototypes our methods could help reduce costs and expedite development of these technologies, which themselves could have important conservation applications.
